# Stages of objective memory impairment are associated with accelerated brain aging

**DOI:** 10.1038/s41598-026-41282-z

**Published:** 2026-04-09

**Authors:** Birthe Kristin Flo, Stavros Skouras, Anna Maria Matziorinis, Tobias Bashevkin, Christian Gaser, Stefan Koelsch

**Affiliations:** 1 Department of Biological and Medical Psychology, University of Bergen, Bergen, Norway; 2 Faculty of Medicine, University of Geneva, Geneva, Switzerland; 3 Department of Neurology, Friedrich Schiller University Jena, Jena, Germany

**Keywords:** Alzheimer’s disease, BrainAGE, Stages of objective memory impairment, MRI, Biomarkers, Neurology, Neuroscience

## Abstract

Reliable staging of early memory decline is essential for identifying individuals at risk for Alzheimer’s disease. The Stages of Objective Memory Impairment (SOMI) framework provides a clinically scalable tool for characterizing episodic memory loss, yet its neurobiological validity remains underexplored. Recent advances in plasma biomarkers (e.g., p-tau217, p-tau181, Aβ42/40) offer emerging blood-based alternatives to CSF and PET, although their diagnostic implementation continues to evolve. Here, we examine whether SOMI stages reflect widespread brain aging, as indexed by BrainAGE, a structural MRI–based biomarker quantifying deviation from normative aging trajectories. While previous studies have linked SOMI to hippocampal atrophy and tau pathology, no study to date has examined its association with a global MRI-derived biomarker of systemic brain aging. In a well-characterized cohort of 119 older adults on the Alzheimer’s disease continuum, we evaluated whether higher SOMI stages were linked to elevated BrainAGE scores and examined whether this association remained significant after adjusting for age, sex, education, and hippocampal volume. Higher SOMI stages were robustly associated with elevated BrainAGE scores, indicating accelerated neurobiological aging. This relationship remained significant after adjusting for covariates and was confirmed in sensitivity analyses. Notably, a marked discontinuity in BrainAGE emerged between SOMI stages 0–2 and 3–5, aligning with the theoretical transition from retrieval to storage impairment, long recognized as a turning point in prodromal Alzheimer’s disease. These findings validate SOMI as a low-cost, non-invasive behavioral marker of systemic brain health. By linking cognitive staging to global neuroimaging biomarkers, our study supports SOMI’s translational utility for large-scale screening, clinical trial stratification, and early intervention planning in Alzheimer’s disease.

**Supplementary Information:**

The online version contains supplementary material available at 10.1038/s41598-026-41282-z.

## Introduction

Alzheimer’s disease (AD) is a progressive neurodegenerative disorder and the leading cause of dementia worldwide^1^. Despite substantial advances in biomarker discovery, a critical implementation gap persists: although cerebrospinal fluid (CSF) analysis and amyloid-PET imaging reliably indicate AD pathology^2,3^, their cost, invasiveness, and limited accessibility render them impractical for large-scale, population-based screening^4,5^. Recent advances in plasma biomarkers such as p-tau217, p-tau181, and Aβ42/40 provide less invasive methods for detecting AD pathology, though their implementation and validation continue to evolve^6,7^. This constraint hampers efforts to identify at-risk individuals during the early, preclinical window, which is precisely the stage when lifestyle modifications and emerging disease-modifying interventions may be most effective. There is therefore an urgent need for alternative tools that are both scalable and biologically informative, that is, tools capable of bridging the gap between advanced biomarker science and practical clinical detection.

In this context, two complementary approaches have gained increasing attention. The Stages of Objective Memory Impairment (SOMI) framework offers a clinically grounded method to stage episodic memory decline (the hallmark cognitive symptom of prodromal AD) based on performance in the Free and Cued Selective Reminding Test (FCSRT)^8^. The FCSRT combines controlled encoding with semantic cue–based retrieval, allowing a clear distinction between retrieval deficits (where cues normalize recall) and storage deficits (where recall remains impaired even with cueing). SOMI stages (0–5) map these qualitative differences, with SOMI-5 later added to capture more severe impairment.

Prior studies have linked SOMI stages to key AD-related pathologies, including amyloid and tau deposition as well as structural atrophy in the hippocampus, entorhinal cortex, and inferior temporal regions (e.g., ^8–12^). Stage-dependent volume reductions of 5–7% in medial temporal lobe structures have been observed, and SOMI has demonstrated predictive utility in longitudinal studies^11^. Notably, the SOMI framework is designed to capture a qualitative transition in memory impairment, from retrieval difficulties in the earlier stages (SOMI 0–2) to actual storage failure at SOMI-3 and beyond. This retrieval-to-storage shift is clinically meaningful, as it marks the onset of the amnestic syndrome characteristic of Alzheimer’s disease and reflects a turning point in underlying neuropathological burden. Consistent with this, prior studies report steeper tau pathology and medial temporal atrophy emerging at SOMI-3.

In parallel, Brain Age Gap Estimation (BrainAGE) has emerged as a powerful neuroimaging biomarker of individual brain health. Derived from structural MRI using voxel-based morphometry (VBM) and machine learning, BrainAGE quantifies the deviation between a person’s chronological and brain-predicted age, yielding a summary metric of whole-brain atrophy^13,14^. Elevated BrainAGE has been associated with increased risk of conversion from MCI to AD and has, in some studies, outperformed classical region-based volumetric measures^15,16^. Unlike traditional single-region indices, BrainAGE reflects distributed neurodegenerative processes, aligning with the multifocal pathology of early-stage AD.

While SOMI provides a clinically intuitive staging of memory dysfunction and BrainAGE offers a biologically sensitive index of systemic brain aging, it remains unknown whether the two converge. Does behavioral evidence of early episodic memory impairment (as captured by SOMI) correspond to an accelerated BrainAGE signature? Addressing this question fills a critical gap in the current literature: it would help establish SOMI not only as a cognitive staging tool, but also as a proxy for global neurobiological aging in preclinical AD. While previous studies have linked SOMI to hippocampal atrophy and tau pathology, no study to date has examined its association with a global MRI-derived biomarker of systemic brain aging.

In the present study, we test whether SOMI stages predict individual differences in BrainAGE. Such a finding would have immediate translational implications: it would position SOMI as a low-cost, non-invasive behavioral indicator of neurodegenerative burden, and strengthen its role in screening, risk stratification, and clinical trial design in the context of Alzheimer’s disease.

## Materials and methods

### Participants

Participants were drawn from the ALMUTH study (*Alzheimer & Music Therapy*; for protocol details, see Flo et al.^17^), a multicenter randomized controlled trial designed to assess music therapy interventions in individuals with or at risk for Alzheimer’s disease. The present cross-sectional analysis focuses exclusively on baseline data, collected prior to the onset of any intervention.

Participants were recruited through outpatient memory clinics and community outreach in Bergen, Norway. Eligibility criteria for the parent study included the presence of subjective cognitive complaints, a Mini-Mental State Examination (MMSE) score above 17, and general health sufficient to permit participation. Exclusion criteria comprised non-AD dementia, significant cardiovascular or neurological disease, history of traumatic brain injury, major psychiatric conditions, sensory deficits that could interfere with testing, and any contraindications to MRI. In addition, individuals with atypical SOMI profiles (severe retrieval deficits without storage impairment) were excluded from the current analysis.

A total of 152 participants were initially screened. Of these, 119 individuals completed high-quality structural MRI and were included in the final analysis sample. All participants provided written informed consent in accordance with the Declaration of Helsinki. The study was approved by the Regional Committees for Medical and Health Research Ethics (REC West; ref. 2018/206).

### SOMI classification

Episodic memory performance was staged using the *Stages of Objective Memory Impairment* (SOMI) framework, based on the picture version of the Free and Cued Selective Reminding Test with immediate recall (pFCSRT + IR). The SOMI system defines six sequential stages (SOMI-0 through SOMI-5) according to thresholds in free and total recall scores (see Table 1), and is designed to capture the gradual progression of memory impairment typical of prodromal Alzheimer’s disease. While SOMI-0 to SOMI-4 are part of the original framework^8^, SOMI-5 was later introduced to accommodate individuals with more pronounced memory deficits^18^. Prior research has demonstrated strong associations between SOMI stages and established AD biomarkers, including amyloid, tau, and medial temporal lobe atrophy^9,11^.

For 16 early-enrolled participants who had a clinical diagnosis of Alzheimer’s disease and were assessed before the implementation of the pFCSRT + IR, SOMI stages were estimated using MMSE scores. Although SOMI was originally developed for preclinical and prodromal AD, SOMI-5 was subsequently introduced to accommodate more severe memory impairment and has been applied in neuropathology-validated studies. Because the aim of the present study was to examine brain correlates across the full spectrum of memory impairment, rather than to diagnose dementia, these participants were included to preserve the continuity of the SOMI staging distribution. Based on the empirical distribution of MMSE scores across SOMI stages, a cut-off of MMSE < 22 was used to distinguish SOMI-5 from SOMI-4. Importantly, we will show in the Results that their inclusion did not bias the results, as demonstrated by a sensitivity analysis excluding these 16 participants, which yielded virtually identical findings (see Results).

**Table 1:** SOMI classification criteria.

SOMI	Free recall scores	Total recall scores	Years to diagnosis: mean (SD)	Class of memory impairment
0 No Memory Impairment	> 30	> 46	7.05 (2.80)	None detected by pFCSRT + IR
1 Subtle Retrieval Impairment	25–30	> 46	4.89 (2.48)	Free recall declines at a constant rate. Storage is preserved.
2 Moderate Retrieval Impairment	20–24	> 46	4.03 (2.62)	Rate of free recall decline doubles. Executive dysfunction accelerates. Storage is preserved.
3 Subtle Storage Impairment	any	45–46	2.09 (1.91)	Cuing fails to normalize total recall.
4 Significant Storage Impairment compatible with dementia	any	33–44	0.86 (1.30)	Intellectual decline accelerates heralding ADL impairment.
5 Moderate Episodic Memory Impairment	any	≤ 32		SOMI 5 was added to accommodate participants with moderate episodic memory impairment.

Criteria and years to diagnosis is derived from Grober et al.^18^

### Neuropsychological assessments

Participants completed a standardized battery of cognitive, functional, and psychosocial assessments. The battery included: the Subjective Cognitive Decline Questionnaire (SCD-Q^19^), consisting of a subscale answered by the participant (MyCog) and an informant-answered subscale (TheirCog), the picture version of the Free and Cued Selective Reminding Test with Immediate Recall (pFCSRT+IR^20^), the Mini Mental State Examination (MMSE^21,22^, Instrumental and Physical Activities of Daily Living (I-ADL, P-ADL^23^, Geriatric Depression Scale (GDS^24^, and a Short Physical Performance Battery (SPPB^25^.

### MRI acquisition and preprocessing

All participants underwent structural magnetic resonance imaging (MRI) using a 3T GE Discovery MR750 scanner (General Electric Medical Systems, Milwaukee, WI, USA) equipped with a 32-channel head coil. High-resolution T1-weighted anatomical images were acquired with a sagittal 3D fast spoiled gradient-echo (FSPGR) sequence. Acquisition parameters were as follows: repetition time (TR) = 6.9 ms, echo time (TE) = 3.0 ms, inversion time = 450 ms, flip angle = 12°, slice thickness = 1.0 mm, in-plane field of view (FOV) = 25.6 × 25.6 cm², matrix size = 256 × 256. The scan duration was approximately 9 min.

Preprocessing was performed using the Computational Anatomy Toolbox (CAT12) within SPM12 (Wellcome Centre for Human Neuroimaging, London, UK), running under MATLAB R2021a. Standard steps included bias field correction, skull stripping, spatial normalization to MNI template space, and segmentation into gray matter, white matter, and cerebrospinal fluid compartments. Gray matter maps were registered using an affine registration and smoothed with a 4 mm and 8 mm full-width-at-half-maximum kernel and further resampled to a spatial resolution of 4 mm and 8 mm.

To reduce data dimensionality and prevent collinearity, principal component analysis (PCA) was performed using the “Matlab toolbox for Dimensionality Reduction” (https://lvdmaaten.github.io/drtoolbox/). This step yielded orthogonal components that support dimensionality reduction and reduce collinearity, consistent with prior recommendations for brain age estimation frameworks^26^.

### BrainAGE estimation

Biological brain age was estimated using the pretrained BrainAGE model developed by Franke and Gaser and implemented in the CAT12 toolbox. This model is based on a relevance vector regression algorithm trained on a large reference sample of cognitively healthy individuals across the adult lifespan^14,26^. BrainAGE was defined as the difference between predicted brain age and chronological age (BrainAGE = predicted age – chronological age), with positive values indicating accelerated neurobiological aging.

The BrainAGE algorithm is trained on voxelwise gray- and white-matter tissue probability maps derived from structural MRI, enabling the model to capture complex multivariate patterns across the whole brain rather than relying on single-region measures such as hippocampal volume. Prior to model training, dimensionality reduction using principal component analysis (PCA) is applied to reduce computational demands, increase robustness, and minimize overfitting. Relevance vector regression is then used to learn age-typical structural patterns, and the resulting model is applied to new MRI data to estimate individual brain age^27^.

To reduce potential domain-shift effects, a linear age-trend correction (a standard step within the BrainAGE framework) was applied based on control subjects from the current cohort. Specifically, predicted brain age was regressed on chronological age in the control group, and the resulting slope and intercept were used to adjust BrainAGE estimates for all participants within the present dataset. This procedure reduces systematic age-dependent bias without retraining the BrainAGE model, while preserving relative between-individual and between-group differences and improving comparability within the cohort.

Our BrainAGE model was calibrated on healthy adults, who on average show a BrainAGE close to zero (reported in Matziorinis et al.^28^). Accordingly, deviations from zero in the present sample are interpreted as reflecting divergence from normative whole-brain aging trajectories, while acknowledging that residual model and domain-shift—related variance cannot be fully excluded.

In summary, the BrainAGE score provides an estimate of accelerated or decelerated brain aging expressed in biologically interpretable units (years).

### Statistical analyses

All statistical analyses were conducted using IBM SPSS Statistics (Version 29). To examine the association between episodic memory staging and biological aging, we first computed a Spearman rank-order correlation between SOMI stage and BrainAGE scores.

Next, we constructed a general linear model (GLM) to test whether SOMI stage significantly predicted BrainAGE, while controlling for chronological age, gender, and years of education. A second model additionally included bilateral hippocampal volume (adjusted for intracranial volume) to examine whether the SOMI–BrainAGE association persisted beyond medial temporal lobe atrophy. All assumptions for linear modeling were assessed and met.

To confirm that our results were not driven by participants with approximated SOMI classifications, we conducted a sensitivity analysis excluding the 16 individuals whose stages were derived from MMSE scores. Results remained consistent with the primary analyses (see Results). Additionally, we explored whether the association between SOMI and BrainAGE reflects a continuous trend or a categorical shift at the onset of storage impairment. For this purpose, we conducted an independent-samples t-test comparing BrainAGE scores between a lower SOMI group (stages 0–2) and a higher SOMI group (stages 3–5).

## Results

### Sample characteristics

Participant characteristics stratified by SOMI stage are summarized in Table 2. Mean age increased progressively across stages, ranging from 66.08 years (SD = 11.64) in SOMI 0 to 75.14 years (SD = 4.18) in SOMI 5. The proportion of female participants varied across groups, with the highest in SOMI 0 (61.3%) and the lowest in SOMI 2 (31.6%). Years of education were relatively stable across stages, with group means between 13.00 and 15.04 years. As expected, BrainAGE scores increased with SOMI stage, reflecting a widening gap between brain-predicted and chronological age in more advanced stages. Neuropsychological performance declined across stages, with lower scores on the FCSRT and MMSE in individuals with higher SOMI stages. Subjective cognitive complaints, particularly informant-based ratings (TheirCog), also increased with advancing impairment. Functional ability, assessed via IADL and PADL, remained relatively high across the sample, though minor declines were observed in later stages. Depressive symptoms (GDS) varied without a consistent trend, and physical performance (SPPB) remained largely preserved. Formal comparisons across groups indicated significant differences in variables such as age, MMSE, and FCSRT performance, whereas other characteristics (such as education) did not differ significantly across SOMI stages (see Table 2 for details).

**Table 2:** Sample characteristics by SOMI stage.

Variable	SOMI 0 (*n* = 30)	SOMI 1 (*n* = 27)	SOMI 2 (*n* = 19)	SOMI 3 (*n* = 9)	SOMI 4 (*n* = 16)	SOMI 5 (*n* = 18)	Group Comparison
Age (years)	65.60 (10.78)	70.41 (11.06)	72.05 (10.55)	76.89 (6.09)	73.94 (8.54)	74.78 (4.93)	H(5) = 15.26, *p* = .009
% Female	63.3%	41.4%	31.6%	66.7%	50.0%	38.9%	χ²(5) = 7.33, *p* = .197
Years of Education	14.77 (3.16)	14.06 (2.45)	14.37 (3.08)	11.75 (4.77)	14.06 (3.42)	14.83 (3.37)	F(5, 112) = 1.29, *p* = .272
BrainAGE	2.97 (4.05)	3.00 (4.06)	4.59 (3.54)	7.37 (3.98)	8.32 (4.00)	9.04 (3.13)	F(5, 113) = 10.22, *p* < .001
FCSRT FR	33.80 (2.59)	27.41 (1.60)	22.32 (1.49)	17.33 (5.32)	14.44 (6.48)	2.89 (3.26)	H(5) = 94.02, *p* < .001
FCSRT TR	47.90 (0.31)	47.74 (0.45)	47.84 (0.38)	45.78 (0.44)	40.11 (3.89)	16.33 (10.37)	H(5) = 79.88, *p* < .001
FCSRT DR	12.53 (1.53)	11.33 (1.62)	9.16 (2.17)	7.11 (1.45)	5.67 (3.71)	0.56 (0.73)	H(5) = 67.78, *p* < .001
MMSE	28.43 (1.57)	28.00 (2.04)	27.63 (2.27)	25.56 (2.60)	24.69 (2.44)	18.83 (2.75)	H(5) = 64.31, *p* < .001
SCD (MyCog)	12.43 (4.68)	11.96 (4.93)	12.68 (4.12)	10.78 (5.40)	8.33 (5.36)	9.00 (3.27)	F(5, 95) = 1.77, *p* = .126
SCD (TheirCog)	7.48 (4.72)	10.11 (5.88)	9.94 (5.20)	14.17 (6.05)	13.44 (5.43)	17.57 (3.60)	F(5, 76) = 5.50, *p* < .001
IADL	8.00 (0.95)	8.74 (1.40)	8.68 (1.57)	8.22 (1.56)	11.25 (5.34)	13.39 (4.73)	H(5) = 39.55, *p* < .001
PADL	6.20 (0.55)	6.12 (0.33)	6.26 (0.81)	6.33 (0.71)	6.81 (1.60)	6.89 (1.28)	H(5) = 11.49, *p* = .043
GDS	6.63 (4.99)	7.70 (6.04)	6.37 (4.63)	7.00 (5.00)	6.88 (5.14)	5.44 (4.36)	H(5) = 1.84, *p* = .871
SPPB	10.53 (1.78)	10.56 (1.74)	10.37 (1.95)	9.67 (2.12)	10.31 (2.58)	9.44 (2.26)	H(5) = 6.87, *p* = .231

Values represent Mean (standard deviation). Gender values reflect the proportion of female participants in each group. Group comparisons were conducted using one-way ANOVA, Kruskal–Wallis H tests, or Chi-squared tests as appropriate. Abbreviations: BrainAGE , brain age gap estimate; FCSRT FR , free and cued selective reminding test – free recall; FCSRT TR , free and cued selective reminding test – total recall; FCSRT DR , free and cued selective reminding test – delayed recall; MMSE , mini-mental state examination; SCD (MyCog), subjective cognitive decline – self-report; SCD (TheirCog), subjective cognitive decline – informant report; IADL , Instrumental activities of daily living; PADL , physical activities of daily living; GDS , geriatric depression scale; SPPB , short physical performance Battery.

**Table 3:** Associations between SOMI stage and MRI-derived markers of brain aging and hippocampal volume.

Outcome	Analysis	Effect size	*p* value	Covariates
BrainAGE	Spearman correlation (vs. SOMI stage)	ρ = 0.53	< 0.001	–
BrainAGE	GLM (SOMI stage predicting BrainAGE)	partial η² = 0.273	< 0.001	Age, sex, education
BrainAGE	GLM (SOMI stage predicting BrainAGE)	partial η² = 0.171	0.002	Age, sex, education, hippocampal volume
BrainAGE	Group comparison (SOMI 0–2 vs. 3–5)	Cohen’s d = 1.32	< 0.001	–
Left hippocampus	Spearman correlation (vs. SOMI stage)	ρ = −0.47	< 0.001	Volume adjusted for ICV
Right hippocampus	Spearman correlation (vs. SOMI stage)	ρ = −0.45	< 0.001	Volume adjusted for ICV

All analyses were conducted in *n* = 119 participants unless otherwise stated. Data are shown as effect sizes for unadjusted correlations and covariate-adjusted models. ICV = intracranial volume. Full model statistics are provided in Supplementary Tables S1–S2.

### Association between SOMI stage and BrainAGE

**Fig. 1:**
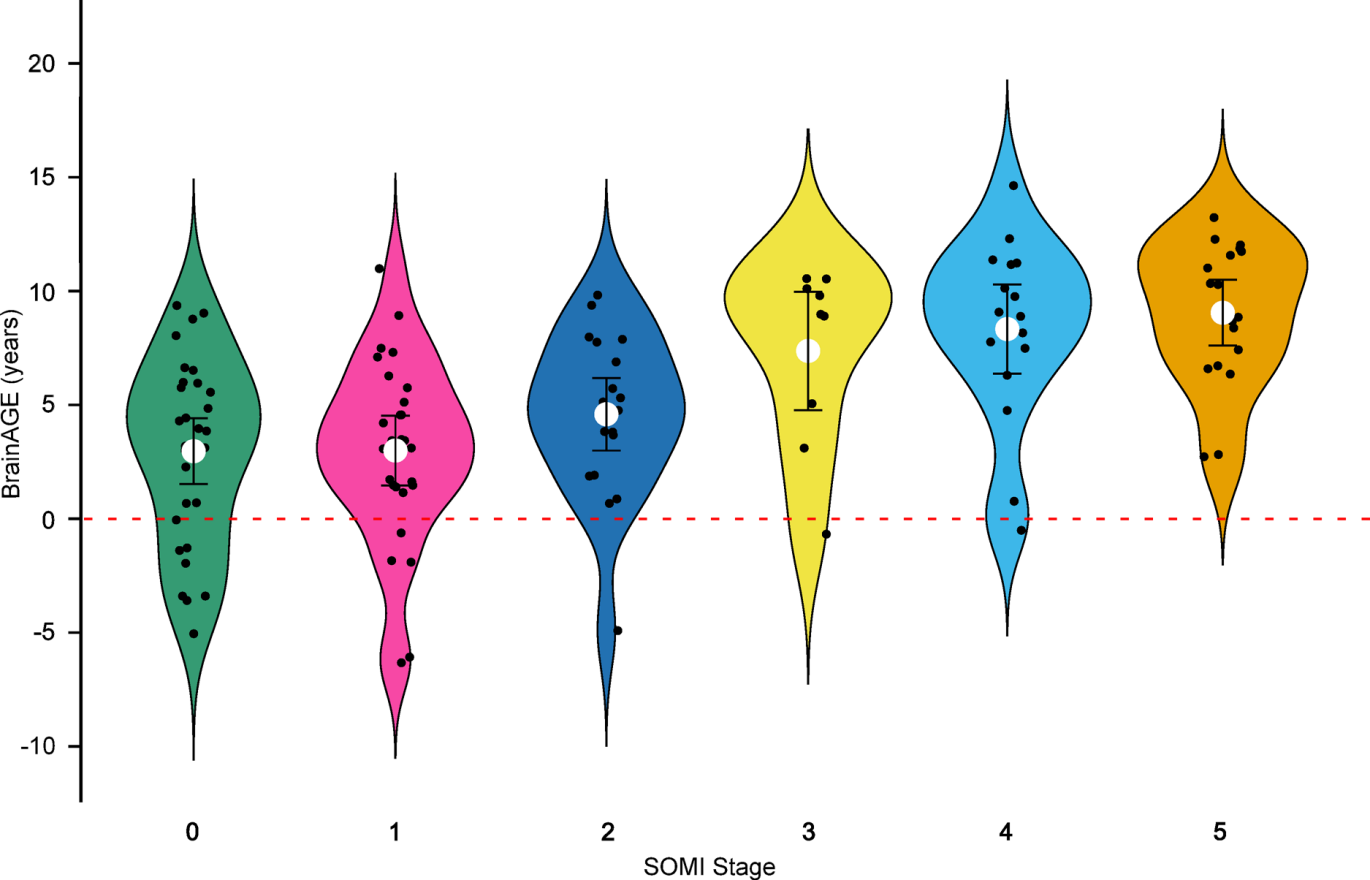
BrainAGE distributions across SOMI stages.

A Spearman rank-order correlation revealed a significant positive association between SOMI stage and BrainAGE, ρ(117) = 0.53, *p* < .001. This suggests that more advanced stages of objective memory impairment are associated with greater deviations from normative brain aging trajectories, i.e., accelerated neurobiological aging. This pattern is illustrated in Fig. 1.

To assess the robustness of this relationship while adjusting for relevant covariates, a general linear model (GLM) was constructed. The overall model was statistically significant, *F*(13, 104) = 8.05, *p* < .001, explaining 50.2% of the variance in BrainAGE (*R²* = 0.502, adjusted *R²* = 0.439). SOMI stage remained a significant positive predictor of BrainAGE, *F*(5, 104) = 7.81, *p* < .001, partial *η*² = 0.273. Chronological age also emerged as a significant predictor, *F*(1, 104) = 29.50, *p* < .001, partial *η*² = 0.221 (see Table S1 for full GLM statistics). In contrast, gender (*p* = .95) and years of education (*p* = .75) were not significant predictors.

To verify that this effect was not driven by SOMI approximations based on MMSE, a sensitivity analysis was conducted excluding the 16 participants with estimated SOMI scores. The association between SOMI and BrainAGE remained statistically significant and comparable in magnitude in this reduced sample (*n* = 103), Spearman’s ρ = 0.459, *p* < .001; multiple regression: β = 0.34, *p* < .001, Adjusted R² = 0.417. These findings confirm the robustness of the primary results.

### Stepwise increase in BrainAGE between SOMI categories

While the overall rank-order correlation between SOMI stage and BrainAGE was significant (ρ(117) = 0.53, *p* < .001), inspection of the group means suggested that the relationship may not be strictly linear. BrainAGE scores showed a modest increase across the lower SOMI stages (0–2), followed by a marked elevation from SOMI-3 onwards (see Table 2). To formally test this pattern, we categorized participants into a lower-impairment group (SOMI 0–2; *n* = 76) and a higher-impairment group (SOMI 3–5; *n* = 43).

An independent-samples *t*-test revealed a significant difference in BrainAGE between the two groups with large effect size, *t*(117) = 6.90, *p* < .001, *d* = 1.32. Participants in the lower SOMI group had a mean BrainAGE of 3.38 years (*SD* = 3.94), compared to 8.42 years (*SD* = 3.62) in the higher group. In contrast, Spearman correlations within each group were not statistically significant: *ρ* = 0.14 (*p* = .23) for SOMI 0–2, and *ρ* = 0.15 (*p* = .34) for SOMI 3–5. These findings suggest that the significant SOMI–BrainAGE relationship is primarily driven by a categorical shift at the transition to storage impairment, rather than by a gradual, stage-wise progression across the full SOMI continuum.

### Association between SOMI stage and hippocampal volume

Correlational analyses confirmed that SOMI stage was significantly associated with medial temporal atrophy. Specifically, SOMI stage negatively correlated with left hippocampal volume, ρ(117) = − 0.47, *p* < .001, and with right hippocampal volume, ρ(117) = − 0.45, *p* < .001.

To examine whether the SOMI–BrainAGE association extended beyond hippocampal degeneration, we conducted a second GLM that included bilateral hippocampal volume (adjusted for intracranial volume) as an additional covariate. This model remained statistically significant, *F*(14, 103) = 9.77, *p* < .001, explaining 57.0% of the variance in BrainAGE (adjusted *R*² = 0.512). Crucially, SOMI stage remained a significant predictor of BrainAGE even after accounting for hippocampal volume, *F*(5, 103) = 4.24, *p* = .002, partial *η*² = 0.171. These results suggest that SOMI stages reflect widespread cortical atrophy and systemic brain aging not fully explained by hippocampal volume alone (see Table S2 for full GLM statistics). A summary of the main associations between SOMI stage, BrainAGE, and hippocampal volume is provided in Table 3.

## Discussion

The present study demonstrates a robust association between the Stages of Objective Memory Impairment (SOMI) and BrainAGE, an MRI-based index quantifying deviation from normative brain aging trajectories. Individuals at more advanced SOMI stages exhibited significantly higher BrainAGE scores, indicating that greater objective memory impairment is associated with more pronounced global structural brain aging. Importantly, this relationship remained significant after adjustment for chronological age, sex, education, and hippocampal volume, and was confirmed in sensitivity analyses excluding participants with estimated SOMI classifications. Together, these findings indicate that SOMI is biologically informative with respect to MRI-derived measures of brain aging.

Previous studies have linked SOMI to amyloid and tau pathology as well as to regional atrophy within medial temporal lobe structures. The present findings extend this work by demonstrating that SOMI is associated with a multivariate index derived from whole-brain structural patterns rather than from a single anatomical region. Because BrainAGE integrates distributed gray- and white-matter information, it captures deviations from typical aging trajectories at the level of whole-brain structure. The observed SOMI–BrainAGE association therefore suggests that behavioral staging of episodic memory impairment corresponds to distributed structural brain alterations detectable with conventional MRI.

### Early-stage heterogeneity in brain aging across SOMI stages

Substantial inter-individual variability in BrainAGE was observed within the early SOMI stages (0–2), in which objective memory performance remains largely preserved. In these stages, individual BrainAGE estimates spanned approximately a decade or more, indicating marked heterogeneity in underlying brain aging among individuals with subjective cognitive complaints. While some contribution of measurement uncertainty inherent to brain age estimation cannot be excluded, this variability is consistent with prior observations of heterogeneous neurobiological trajectories in individuals at risk for Alzheimer’s disease. Such heterogeneity may reflect differences in underlying pathology burden, brain reserve, or disease progression stage.

Indeed, elevated and variable BrainAGE estimates have been reported previously in individuals with subjective cognitive decline, even in the absence of measurable objective impairment. In this context, variability in early SOMI stages may indicate sensitivity of BrainAGE to subtle neurobiological alterations that precede overt memory deficits.

Notably, participants classified as SOMI-0 and SOMI-1 demonstrated modestly elevated BrainAGE scores despite intact performance on the Free and Cued Selective Reminding Test. Because the BrainAGE model is calibrated such that cognitively healthy adults show mean values close to zero on average^28^, this elevation is unlikely to reflect model artifact alone. Rather, it suggests that individuals with subjective cognitive concerns may already exhibit subtle but biologically meaningful deviations from normative brain aging, even before objective memory deficits become detectable. This interpretation is consistent with recent findings reporting increased BrainAGE in subjective cognitive decline cohorts, including results from the REMEMBER study^29^.

### Stage-dependent patterns and the transition to storage impairment

Although the overall association between SOMI stage and BrainAGE was significant, the relationship was characterized by a threshold-like pattern rather than a linear increase across all stages. BrainAGE values showed a relatively modest increase across SOMI stages 0–2, followed by a marked elevation from SOMI-3 onward. Formal analyses confirmed a large difference in BrainAGE between retrieval-based stages (SOMI 0–2) and stages characterized by storage impairment (SOMI 3–5), whereas correlations within each subgroup were not statistically significant.

This pattern is consistent with the conceptual design of the SOMI framework, which emphasizes a qualitative transition from retrieval deficits to genuine storage impairment. Importantly, this does not imply that neurobiological changes are absent in earlier stages; rather, pathological processes may accumulate gradually across the disease continuum, while clinically meaningful storage failure emerges at a later point. The sharp increase in BrainAGE at SOMI-3 provides independent neurobiological support for this theoretical inflection point and aligns with previous reports of accelerated tau pathology and medial temporal atrophy at the onset of storage impairment.

Together with the observed variability and modest BrainAGE elevations in early SOMI stages, these findings support a model in which neurobiological changes evolve progressively, while SOMI stages capture clinically meaningful transitions in episodic memory function. As the present analyses are cross-sectional, longitudinal studies are required to determine whether this apparent threshold reflects within-individual acceleration of brain aging over time.

### SOMI associations beyond hippocampal volume

The persistence of the SOMI–BrainAGE association after controlling for hippocampal volume indicates that SOMI staging corresponds to structural brain aging patterns that extend beyond medial temporal lobe atrophy. While the FCSRT is sensitive to hippocampal dysfunction, the present results suggest that SOMI reflects broader structural alterations detectable at the whole-brain level. This should not be interpreted as evidence for network-level breakdown or systemic degeneration, but rather as an association between memory impairment severity and distributed structural aging patterns not fully explained by hippocampal volume alone.

From a clinical perspective, this supports the utility of SOMI as a behavioral staging tool that corresponds to global MRI-derived measures of brain aging, while remaining feasible for large-scale, low-cost assessment.

### Strengths, limitations, and future directions

Several strengths of this study warrant emphasis, including the use of a validated cognitive staging framework, standardized MRI acquisition and preprocessing, and a BrainAGE model with demonstrated robustness across cohorts. Nonetheless, important limitations must be acknowledged. First, the cross-sectional design precludes inference about causal or temporal relationships between memory decline and brain aging. Second, although sensitivity analyses demonstrated that results were virtually unchanged when excluding participants with MMSE-estimated SOMI classifications, the inclusion of estimated stages remains a methodological consideration. Third, BrainAGE models are trained on healthy reference samples, and although deviations from zero reflect biologically meaningful divergence from normative aging, residual domain-shift effects cannot be entirely excluded. Finally, all participants reported subjective cognitive complaints, which may limit generalizability to asymptomatic populations.

Future studies integrating longitudinal designs, multimodal neuroimaging (e.g., diffusion MRI or functional connectivity), and molecular biomarkers will be essential to clarify the temporal dynamics and biological specificity of SOMI–BrainAGE associations.

## Conclusion

Our findings demonstrate that SOMI stages are significantly associated with BrainAGE, a well-established MRI-derived index of deviation from normative brain aging trajectories. The observed discontinuity in BrainAGE between SOMI stages 0–2 and 3–5 parallels previously reported stage-dependent changes in tau pathology and medial temporal atrophy^9,10,30^, supporting the biological relevance of this cognitive staging framework. This transition corresponds to the shift from retrieval difficulties to storage impairment—a defining feature of Alzheimer’s-type memory decline—and was associated with marked differences in BrainAGE in the present sample.

The SOMI–BrainAGE association remained robust in sensitivity analyses and after adjustment for hippocampal volume, indicating that SOMI reflects structural brain aging patterns not fully captured by medial temporal lobe atrophy alone. Together, these findings support SOMI as a clinically scalable and biologically informative tool for characterizing memory impairment severity and its neurobiological correlates, with potential relevance for research on early Alzheimer’s disease.

Figure legend: Violin plots show the distribution of BrainAGE values across SOMI stages 0 to 5. White circles denote group means, with vertical lines indicating 95% confidence intervals. Black dots represent individual participants. The red dashed line indicates BrainAGE = 0, where predicted and chronological age align. The figure illustrates a progressive increase in BrainAGE with advancing SOMI stage. Notably, BrainAGE scores rise sharply at the transition from SOMI-2 to SOMI-3, consistent with the onset of storage impairment and supporting the biological validity of this cognitive staging threshold.

## Supplementary Information

Below is the link to the electronic supplementary material.

Supplementary Material 1

## Data Availability

The data supporting the findings described can be obtained from the corresponding author upon request.

## Abbreviations

AD: Alzheimer’s disease
Aβ42/40: Amyloid beta 42/40
BrainAGE: Brain age gap estimation
CAT12: Computational anatomy toolbox
CSF: Cerebrospinal fluid
FCSRT: Free and cued selective reminding test
FCSRT DR: Free and cued selective reminding test—delayed recall
FCSRT FR: Free and cued selective reminding test—free recall
FCSRT TR: Free and cued selective reminding test—total recall
FOV: Field of view
FSPGR: Fast spoiled gradient echo
GDS: Geriatric depression scale
GLM: General linear model
IADL: Instrumental activities of daily living
IR: Immediate recall
MCI: Mild cognitive impairment
MMSE: Mini-mental state examination
MRI: Magnetic resonance imaging
MNI: Montreal neurological institute
PADL: Physical activities of daily living
PCA: Principal component analysis
PET: Positron emission tomography
pFCSRT + IR: Picture version of the free and cued selective reminding test with immediate recall
p-tau18: Plasma phosphorylated tau at threonine 181
p-tau217: Plasma phosphorylated tau at threonine 217
REC: Regional Committee for Medical and Health Research Ethics
RVR: Relevance vector regression
SCD: Subjective cognitive decline
SCD-Q: Subjective cognitive decline questionnaire
SOMI: Stages of objective memory impairment
SPM: Statistical parametric mapping
SPPB: Short physical performance battery
TE: Echo time
TR: Repetition time
VBM: Voxel-based morphometry
