# Supplementary material for: Stages of objective memory impairment are associated with accelerated brain aging
Source: Sci Rep. 2026 Apr 9;16:11837. doi: 10.1038/s41598-026-41282-z (PMC13066603; doi:10.1038/s41598-026-41282-z)
Supplement: Supplementary file 1 — Supplementary Material 1 [file 41598_2026_41282_MOESM1_ESM.pdf]

Supplementary Materials

Table S1. General Linear Model Predicting BrainAGE (Model 1: SOMI + age/sex/education)

Predictor	F	p	Partial η²	β (B)	Adjusted R²
Age_Years	29.502	<.001	.221	0.188	.439
Years_Education	0.095	.759	.001	-0.033	.439
SOMI_stage (overall)	7.814	<.001	.273		.439
Gender (overall)	0.003	.953	.000		.439
SOMI_stage × Gender	0.844	.521	.039		.439

Table S1. General linear model (GLM) predicting BrainAGE from SOMI stage, age, gender, and years of education.Values shown are F statistics, p-values, partial η^2, unstandardized regression coefficients (B), and adjusted R^2.

Table S2. General Linear Model Predicting BrainAGE (Model 2: Model 1 + hippocampal volume)

Predictor	F	p	Partial η²	β (B)	Adjusted R²
Age_Years	15.762	<.001	.133	0.138	.512
Years_Education	0.243	.623	.002	-0.050	.512
TotalHippocampalVolume_Adjusted	16.481	<.001	.138	-1899.197	.512
SOMI_stage (overall)	4.238	.002	.171		.512
Gender (overall)	0.014	.908	.000		.512
SOMI_stage × Gender	0.469	.799	.022		.512

Table S2. General linear model (GLM) predicting BrainAGE from SOMI stage, age, gender, years of education, and hippocampal volume (adjusted for intracranial volume). Values shown are F statistics, p-values, partial η^2, unstandardized regression coefficients (B), and adjusted R^2.
